# Editorial: Special Issue “Antenna Design for 5G and Beyond”

**DOI:** 10.3390/s21227745

**Published:** 2021-11-21

**Authors:** Naser Ojaroudi Parchin, Chan Hwang See, Raed A. Abd-Alhameed

**Affiliations:** 1 School of Engineering and the Built Environment, Edinburgh Napier University, Edinburgh EH10 5DT, UK; c.see@napier.ac.uk; 2 Faculty of Engineering and Informatics, University of Bradford, Bradford BD7 1DP, UK; r.a.a.abd@bradford.ac.uk

The demand for high data rate transfer and large capacities of traffic is continuously growing as the world witnesses the development of the fifth generation (5G) of wireless communications with the fastest broadband speed yet and low latency [1]. Widespread coverage, adequate signal quality, and low latency are just a few of the benefits that have sparked interest in 5G networks. The commercialization of 5G communication has already started, as well as initial research into beyond technologies such as 6G [2]. The implementation of 5G technology is either in sub 6 GHz or in the millimetre-wave (MM-Wave) region. As a crucial part of future communication systems, breakthroughs in antenna systems will obviously improve the performance of the entire communication system [3]. Antenna design for 5G and beyond networks should apply careful considerations with some key requirements. Wide frequency coverage, the larger number of low-profile antenna elements, ease of fabrication, and conformity are some of the key parameters for the success of 5G antenna systems [4]. Substantial advances have been made in the design, optimization, and development of new antennas for 5G and beyond. In future antenna systems, two main antenna arrangements are considered, including multiple-input multiple-output (MIMO) and phased array with multiple elements. Multiple antenna elements are used to create increased multiple spatial channels, which leads to the improved data rate of the system [5].

This Special Issue covers various aspects of novel antenna designs for 5G and beyond applications. The featured topic articles in this issue highlight recent advances in designing antenna systems for smartphones, small cells, platform stations, massive MIMO, MM-Wave, front-end, metamaterials, and metasurface applications. In addition, other relevant subjects such as spectral efficiency improvement, K-user MIMO, and multi-mode band-pass filter design are discussed. This Special Issue is a collection of fifteen papers that are briefly explained in the following.

Seyyedesfahlan et al. [6] report on the design of a wideband and multi-port antenna for sub 6 GHz applications. The structure of the single element is composed of a circular radiation disk (with λ/4 diameter) with microstrip-line feeding and a modified triangular-shaped ground plane. It is designed to operate at the frequency range from 2 to 6 GHz. The antenna exhibits sufficient gain and efficiency results. In addition, two- and four-port MIMO configurations of the design with low mutual coupling are studied. The design antennas were fabricated, and their simulation and measurement results show good agreement. The multi-port antenna designs can be used in sub 6 GHz 5G wireless systems.

Ojaroudi Parchin et al. [7] propose a new ultra-wideband (UWB) MIMO antenna system design for future smartphones. It contains four pairs of compact microstrip-fed slot antennas placed at four different edge corners of the smartphone mainboard. The antenna elements provide radiation and polarization diversity, and they operate at a frequency of 2.5–10.2 GHz. Critical characteristics of the antenna elements and MIMO design, such as operation bandwidth, antenna gain and efficiency, and radiation patterns are investigated in detail. The authors also highlight the MIMO performance and channel capacity of the proposed smartphone antenna.

Ojaroudi Parchin et al. [8] demonstrate a new 5G smartphone antenna design with eight modified PIFA elements placed at four corners of the mainboard. It operates at three different frequencies of sub 6 GHz, namely 2.6, 3.6, and 5.8 GHz. In addition, in order to support the MM-Wave 5G spectrum, a compact phased array antenna with a wide bandwidth of 25–31 GHz is introduced for incorporation into a shared board. According to the presented results, quite good characteristics in terms of operation bandwidth, antenna gain, and radiation pattern are observed for the proposed antenna, both with and without the presence of user-head, user-hand, and smartphone components.

Sharaf et al. [9] developed a compact dual-band patch antenna to support 36 and 60 GHz for 5G mobile communications. The antenna configuration contains two electromagnetically coupled patches creating the first and second resonances. Due to its small profile, the proposed patch antenna design can be used either as a single antenna or an element to construct compact MIMO antenna systems. The antenna critical characteristics are assessed through numerical and experimental investigations and good results are observed. In addition, numerical comparisons show that the introduced patch antenna is superior to other published designs.

Dixit et al. [10] introduce a new antipodal Vivaldi array antenna with a 1 × 4 arrangement proposed for 5G MM-Wave applications. A corporate feeding network is applied on the top layer of the Vivaldi array design to feed the elements. It resonates in a wide frequency range of 24.19–40.47 GHz, covering n257~n261 bands of 5G. Using corrugations in the design of resonators, antenna characteristics such as gain level and front-to-back ratio are improved. The antenna exhibits high gain levels (varying from 8 to 13.2 dBi) versus its operation band. It is designed on a Rogers-5880 substrate material with overall dimensions of 24 × 28.8 × 0.254 mm^3^.

Song et al. [11] present a low-cost/low-profile metasurface transmit-array design, fed by a compact antenna array for 28 GHz 5G systems. The employed metasurface contains two metallic layers at different sides of the substrate along with four fixed vias connecting the layers. The transmit-array is composed of several unit cells with different dimensions. In order to optimize the phase distribution on the transmit-array and decrease the side-lobe level, the authors introduced particle swarm optimization. The optimized design achieves 27 dB gain with 11.8% 3 gain bandwidth, −30 dB side-lobe level, and aperture efficiency of 23%, which are improved from the unoptimized design.

Hamid et al. [12] focused on investigating the multibeam characteristics of a negative refractive index-shaped lens with high gain and narrow beamwidth to be operated at upper 5G frequency bands. Two types of negative refractive index lenses, including energy conservation and Abbe’s sine lenses, were designed and their parameters are studied. They exhibit high gains and narrow beamwidth characteristics with 65~66% efficiency results. In addition, both designs provide optimum results for beam scanning up until 40°. The designed negative refractive index-shaped lens offers good characteristics in terms of fundamental properties and can be used in 5G mobile base stations.

Kozlov et al. [13] present a new 1-bit dual-polarized tiled transmit-array with beam-steering function for 5G communication systems. The configuration of a dual-polarized unit cell contains a pair of identical square-ring resonators via a pair of U-shaped feed loops. The proposed is highly flexible and adjustable for adding individual elements and using different feeding types. It offers 160 MHz impedance bandwidth from 5.67 to 5.83 GHz. An example of the proposed design with 10 × 10-element is presented, and its simulation and measurement performances are discussed in detail. The circuit model topology of the proposed beam-steerable transmit-array is discussed.

Ferreira-Gomes et al. [14] demonstrate a small-sized integrated metasurface antenna design with circular-polarized characteristics for 5G MM-Wave communications. Its configuration is composed of a chiral dielectric metasurface and a 2 × 2 arrangement of dielectric cylinders to reach optimized radiation patterns. The designed metasurface antenna is fed using the SIW-to-coax feeding technique. The proposed antenna operates from 25.3 to 31.6 GHz (22.6%) with 11.6% 3 dB axial ratio (AR). The investigated results show promising improvements in antenna gain, AR, and impedance bandwidth. The antenna has a planar structure and can be easily integrated into wireless systems.

Ramírez Arroyave et al. [15] developed a new design technique to minimize the nonlinear distortion and maximize power efficiency for an MM-Wave PIN diode in reconfigurable antennas. This is mainly achieved through the reduction of the mutual coupling between the internal switching and the external feeding ports obtained using a test-set with a nonlinear network analyser, on-wafer probe station, and a test fixture. The nonlinear models are extracted through X-parameter measurements and are validated by S-parameter in the low power signal regime and by harmonic measurements. A particular example of antenna design in 3.5 GHz is demonstrated to verify the minimum nonlinearities method.

Gaya et al. [16] proposed a novel feeding method for dielectric resonator antennas. In the proposed design, a ceramic-based dielectric resonator antenna is fed by a metallic patch structure via a cross-slot aperture on the back layer. Using the cross-slot aperture, the maximum power radiation along the broadside and the antenna gain are improved. In order to achieve the desired impedance match, the slot dimensions are optimized. The antenna is designed to operate at 26 GHz 5G band, and it offers high gain and efficiency characteristics over its operation band. These features make the design suitable to be used for indoor small cell applications.

Iqbal et al. [17] have developed a wideband half-mode substrate-integrated waveguide (HMSIW) filter for 5G front-end applications. In the proposed design, a semi-circular cavity resonator is applied to obtain a wide operation band of 3–7 GHz. In addition, in order to improve the out-of-band response, a U-shaped slot and a pair of L-shaped stubs are employed in the configuration of the design. Wide bandwidth, loss, planar structure, and ease of integration are some attractive features of the 5G filter design. In addition, it is better in terms of insertion loss when compared with other filters reported in the literature. The filter was fabricated on the RT/duroid 5880 substrate and exhibits good measurement results.

Luo et al. [18] present a new method to obtain continuous tuning without resonance blindness in the PIN diode. In this method, after achieving the equivalent impedance of the diodes, these nonlinear properties will be fitted with mathematical expressions. Using the mathematical equations, the PIN diode is modelled to be compatible with implementing co-simulation. Finally, in order to validate the usefulness of the proposed method, the co-simulation and experimental results are carried out at 5 GHz. In this approach, the active metamaterial structure contains two periodical cells with a PIN diode in each unit, inductance chips, and via holes between the top and bottom layers.

In their work, Dicandia and Genovesi [19] focused on improving the spectral efficiency of 5G massive MIMO systems using triangular lattice arrays. The authors investigated the beneficial effects of adopting a triangular lattice on phased arrays with regular and periodic grids. A particular study demonstrates the impact of the antenna array lattice at the system level. A planar array with 64 elements and an arrangement of 8 × 8 is analysed to better emphasize the advantages of the proposed method. The better performance achieved by the triangular lattice array makes it appealing for high-altitude platform station (HAPS) systems.

Yerrapragada and Kelley [20] propose a high-throughput wireless access framework (known as K-User MIMO) for beyond-5G or 6G networks. It drives the multi-user Shannon capacity formula to improve the spectral efficiency of the systems. The authors also define an OFDM frame structure to demonstrate the allocation of time-frequency resources for channel estimation. In addition, the performance of the proposed framework has been compared with related technologies. The introduced framework can cancel interference, demodulate, and maximize capacity through signal separation. It can also facilitate all-to-all communication between access points and mobile devices.

We would like to take this opportunity to appreciate and thank all authors for their excellent contributions and the reviewers for their fruitful comments and feedback. Special appreciation is also extended to the editorial board and editorial office of MDPI *Sensors* journal. We hope that readers will discover new and useful information on antenna design techniques for 5G and beyond applications.
